# Monte Carlo Approach to the Evaluation of Nanoparticles Size Distribution from the Analysis of UV-Vis-NIR Spectra

**DOI:** 10.3390/mi14122208

**Published:** 2023-12-06

**Authors:** Cristiano Lo Pò, Valentina Iacono, Stefano Boscarino, Maria Grazia Grimaldi, Francesco Ruffino

**Affiliations:** 1 Dipartimento di Fisica e Astronomia “Ettore Majorana”, Università di Catania, Via S. Sofia 64, 95123 Catania, Italy; 2 CNR-IMM, Via S. Sofia 64, 95123 Catania, Italy; 3 Research Unit of the University of Catania, National Interuniversity Consortium of Materials Science and Technology (INSTM-UdR of Catania), Viale Andrea Doria 8 and Via S. Sofia 64, 95125 Catania, Italy

**Keywords:** nanoparticles, plasmonic, Monte Carlo, simulations, copper, gold, Mie theory, laser ablation

## Abstract

How nice would it be to obtain the size distribution of a nanoparticle dispersion fast and without electron microscope measurements? UV-Vis-NIR spectrophotometry offers a very rapid solution; however, the spectra interpretation can be very challenging and needs to take into account the size distribution of the nanoparticles and agglomeration. This work suggests a Monte Carlo method for rapid fitting UV-Vis-NIR spectra using one or two size distributions starting from a dataset of precomputed spectra based on Mie theory. The proposed algorithm is tested on copper nanoparticles produced with Pulsed Laser Ablation in Liquid and on gold nanoparticles from the literature. The fitted distribution results are comparable with Transmission Electron Microscope results and, in some cases, reflect the presence of agglomeration.

## 1. Introduction

Nowadays, nanoparticles (NPs) are used in a wide range of applications and devices thanks to the combination of classical and quantum effects in optical, conductive, magnetic properties, and so forth. In particular, copper and gold NPs are widely used in plasmonic devices due to their optical properties in the visible light range. The UV-Vis-NIR spectra of Cu and Au NPs present a typical peak, called plasmonic peak, in a specific wavelength range, depending on the material, that can be used for various devices and applications [1,2,3,4,5,6,7,8,9,10].

The plasmonic peak position and shape strictly depend on the NPs size and shape [11,12], so knowing the particle size distribution is very important. Transmission Electron Microscopy (TEM) analysis gives detailed information about the particle size distribution but does not give appreciable information on the aggregation status, and both the sample preparation and analysis require a long time [13]. Also, sample preparation can alter it, especially for copper and other non-precious metals that can oxidize. Scanning Electron Microscopy (SEM) analysis is faster, and the sample preparation is easier, but a size resolution under ∼10 nm is commonly hard to obtain. UV-Vis-NIR spectrophotometry can perform rapid measurements of particle suspension without altering the sample. In particular, the extinction cross section (given by absorption plus scattering) is related to the particles’ shape and size through Mie scattering theory [11,12,14]. A fitting routine that uses Mie scattering is not easy to implement, and for the few works present in the scientific literature, everyone considers a constant refractive index for the solvent. An efficient fitting routine is proposed in Refs. [13,15] for monodisperse spheroidal gold NPs using the Mie–Gans model. Other approaches of fitting or simulation are proposed in the scientific literature mainly for gold monodispersed NPs or spheroids, like Machine Learning [16], Least Squares Approximation combined with matrix formalism [17], Discrete Dipole Approximation [18] and Effective Medium theories for NP clusters [19].

The aim of this work is to develop a versatile computational method to easily extract metal NPs size distribution from an optical spectrum using a fast Monte Carlo technique (in terms of the number of iterations) that can work both with monodisperse and polydisperse nanoparticles in a wide range of wavelengths (250–1100 nm). In particular, a dataset will be previously computed to fit the experimental data using some physical considerations as an alternative to a mathematical or Machine Learning approach. Firstly, the code is first used on experimental spectra of some copper NPs dispersions produced with Pulsed Laser Ablation in Liquid (PLAL) and then on the gold literature data.

## 2. Materials and Methods

### 2.1. Experimental Section

Copper NPs were synthesized using an Nd:YAG ns-pulsed laser (Quanta-Ray PRO-Series Nd:YAG with wavelength λ = 1064 nm, pulse length = 12 ns, mean power = 5 W, and repetition rate = 10 Hz) with the methodology described in Ref. [20]. A lens (focal length of 10 cm) focused the laser beam on a copper target at the bottom of a Teflon vessel, filled with 8 mL of liquid (acetone, methanol, and ethanol). The ablated mass was measured with a Sartorius M5 microbalance (sensitivity 0.01 mg) by weighting the target before and after the ablation, resulting, respectively, in 0.07 mg, 0.13 mg, and 0.70 mg for acetone, methanol, and ethanol with an accuracy of 0.02 mg.

The obtained NPs colloidal solutions were sonicated for 15 min and optically analyzed with a PerkinElmer LAMBDA 1050+ UV-Vis-NIR Spectrophotometer, measuring the absorbance from 200 nm to 1100 nm. A baseline correction was performed using the measured absorbance of the relative solvent for each solution.

### 2.2. Computational Section

**Figure 1:**
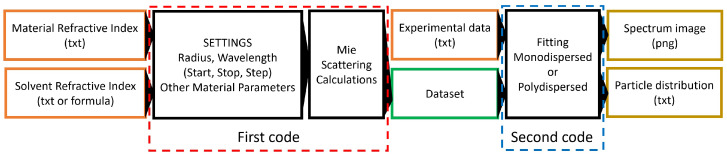
Schematic description of the software developed.

Computational analysis is performed using two codes developed on Wolfram Mathematica 13 software [21]. The overall process is schematically represented in Figure 1.

The first code (Dataset_creation.nb, described in Appendix C), uses the results of the Mie scattering theory to create a dataset containing the spectrum of spherical particles having a different size. The starting point for these simulations is the material’s and medium’s refractive index, which can be easily found in an online database [22] or in the Palik Handbook [23]. Unfortunately, more than ten refractive indexes for copper are available in the literature, in the visible range, with slight differences among them. So the copper refractive index was experimentally evaluated (see Appendix A). This code should work also in the UV region, where the refractive index of the solvents cannot be considered a constant, so their formulas are taken from the online database [22] and reported in Appendix B. A database is computed for each solvent for various radii and wavelength ranges.

**Figure 2:**
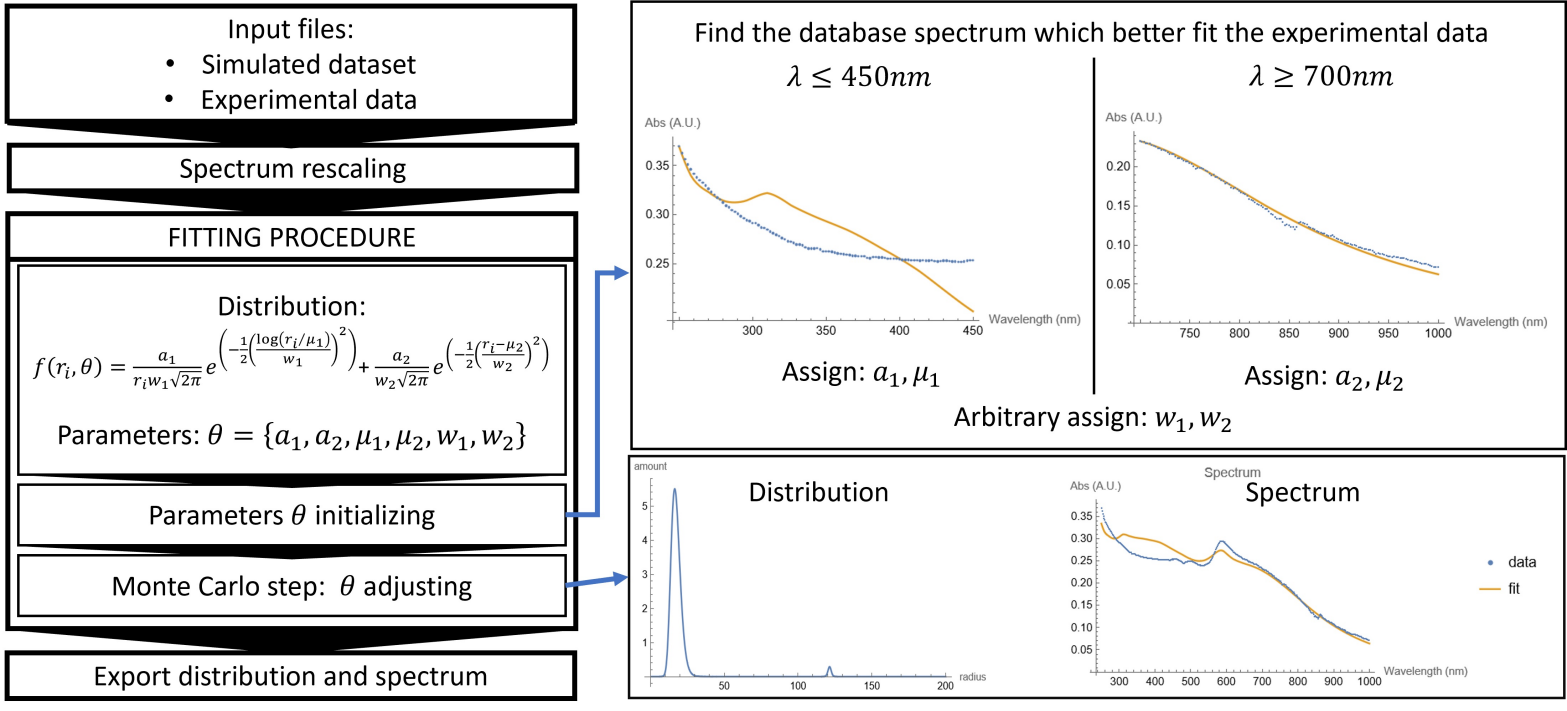
Schematic description of the fitting algorithm with example graphics of polydisperse copper NPs produced in ethanol with PLAL.

The second code comes in two versions: one is for monosdispersed NPs colloidal solution (Mono_Fitting.nb) and the other is for polydispersed ones (Poly_Fitting.nb algorithm presented in Figure 2). Firstly, both the experimental data and cross section from the dataset are acquired. The experimental data are usually reported in arbitrary units, and the computed cross sections present values of the order of magnitude less than $10^{-13}\;m^{2}$. Working with these values is computationally inconvenient; for this reason, they will be rescaled. The total absorption spectrum of a given particle distribution f(r) is obtained by integrating over the full particle radius in the range, but when computed, the integral must be discretized:

(1) $$\sigma_{tot}\left(\lambda \right)=\frac{\int_{0}^{\infty }f\left(r\right)\sigma \left(\lambda ,r\right)dr}{\int_{0}^{\infty }f\left(r\right)dr}\to \frac{\sum_{r_{i}=r_{0}}^{r_{max}}f\left(r_{i}\right)\sigma \left(\lambda ,r_{i}\right)}{\sum_{r_{i}=r_{0}}^{r_{max}}f\left(r_{i}\right)}$$

**Figure 3:**
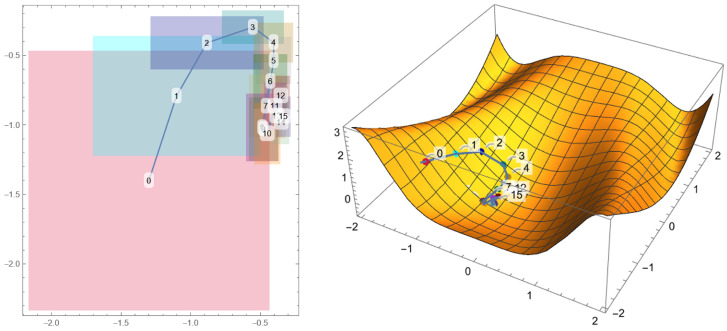
Example of the same Monte Carlo gradient descent implemented on a two-variable function for a schematic visualization. On the left, the numbered boxes indicate the parameter values and the range in which random number generators work. On the right, the same numbered points descend toward the minimum of an example function.

The denominator $\sum_{r_{i}}f\left(r_{i}\right)$ does not depend on the wavelength and acts like a scale parameter, so it is ignored to lighten the computational burden. The colloidal solutions usually are characterized by monodisperse or polydisperse nanoparticles [24]. In the most simple case (monodisperse NPs), the size distribution follows a lognormal distribution:

(2) $$f\left(r_{i},a_{1},\mu_{1},w_{1}\right)=a_{1}\frac{1}{r_{i}w_{1}\sqrt{2\pi }}exp\left(-\frac{1}{2}{\left(\frac{log(r_{i}/\mu_{1})}{w_{1}}\right)}^{2}\right)$$

with three parameters $\theta =\{a_{1},\mu_{1},w_{1}\}$. Polydisperse solutions present at least two separate size distributions. In this case, a lognormal size distribution for smaller particles is used, while for bigger particles or aggregates, a Gaussian size distribution is chosen. This choice is due to the random particle aggregation process [13]. Therefore, the assumption is that the global distribution is:

(3) $$f\left(r_{i},a_{1},a_{2},\mu_{1},\mu_{2},w_{1},w_{2}\right)=a_{1}\frac{1}{r_{i}w_{1}\sqrt{2\pi }}exp\left(-\frac{1}{2}{\left(\frac{log(r_{i}/\mu_{1})}{w_{1}}\right)}^{2}\right)+a_{2}\frac{1}{w_{2}\sqrt{2\pi }}exp\left(-\frac{1}{2}{\left(\frac{r_{i}-\mu_{2}}{w_{2}}\right)}^{2}\right)$$

with six parameters $\theta =\{a_{1},a_{2},\mu_{1},\mu_{2},w_{1},w_{2}\}$. A fitting routine for $f(r_{i},\theta )$ is manually implemented to find the optimal parameter set θ that minimizes the mean squared error (MSE) between the simulation $X_{i}$ and the experimental data $Y_{i}$. The MSE is defined as:

(4) $$MSE=\frac{1}{n}\sum_{i=1}^{n}{\left(X_{i}-Y_{i}\right)}^{2}\to ERROR=\sum_{\lambda_{i}=\lambda_{min}}^{\lambda_{max}}{\left(\sigma_{tot}\left(\lambda_{i}\right)-\sigma_{data}\left(\lambda_{i}\right)\right)}^{2}$$

where $\sigma_{tot}$ contains a sum over all the radii (Equation (1)) and the multiplication factor 1/n is ignored. This new quantity ERROR is proportional to MSE and will be minimized. The fitting routine is divided into three steps:

1. Files reading: Experimental data are acquired, sorted, and normalized. The dataset is acquired at the same wavelengths as the experimental points, and it is also rescaled. This automatically leads to the use of the wavelength range in which both the experimental data and the computed dataset are defined.
2. Assign starting point parameters: choosing the starting point parameters for a function of three or six parameters is crucial. Starting with some random parameters can lead the gradient to descend toward a local minimum without specific physical significance. It is known that “*With four parameters I can fit an elephant, and with five I can make him wiggle his trunk—E. Fermi*” [25]. To pursue this aim, two strategies are followed:
  - Monodisperse NPs: The ERROR is evaluated between the experimental data and every spectrum in the dataset. The spectrum that produces the minimum ERROR gives the starting point for the distribution centroid $\mu_{1}$ and the scale parameter $a_{1}$. This evaluation is performed in a small range (a convenient one can be 400nm≤λ≤700nm because gold and copper have their plasmonic peak within this range).
  - Polydisperse NPs: The ERROR is evaluated between the experimental data and every spectrum in the dataset in two different ranges. Small particles strongly contribute in the UV, so $a_{1}$ and $\mu_{1}$ (lognormal distribution) are assigned by finding the minimum ERROR among the computed spectra for λ≤350nm. Bigger particles and aggregates strongly contribute in the IR, so $a_{2}$ and $\mu_{2}$ (Gaussian distribution) are assigned by finding the minimum ERROR among the computed spectra for λ≥700nm.
  These edge values for λ are purely indicative and can easily be changed in the code to find the optimal starting point for each sample. The initial values of $w_{1}=0.5$ and $w_{2}=3$ are assigned arbitrarily.
3. Monte Carlo step: A cycle where a new set of parameters θ is randomly generated each time within a range of the initial parameter. Whenever the ERROR obtained with the new set of parameters is lower than the initial ERROR, the parameters are updated, and the process is repeated for a fixed number of iterations, but new parameters can now vary in a smaller range than the previous one:
  (5) $$\theta_{j}:=\theta_{j}\left(1+Range\cdot Random\left[-1,1\right]\right)$$
  where Random[−1,1] indicates a random number generated between −1 and 1, and Range=1/(2+Count) is the range in which the new parameter is generated, with Count=0 that increases at every successful parameter update (the symbol := is used to indicate a variable update). A visual representation of this process is given in Figure 3. The relative error associated with each parameter is given by $1/\sqrt{m}$, where *m* is the number of iterations. At the end of the cycle, a plot and a text file are exported.

This approach of separating the theoretical computation (first code) and the curve fitting (second code) represents an alternative to the methods proposed in the literature [13,15], where the cross section is computed time by time. Computing the dataset separately from the fitting allows to use the dataset multiple times without recomputing it. The dataset computing time and the use of computational resources depend on the wavelength range and the radius range. In this work, the Mie scattering theory is used to compute the dataset instead of the Gans theory used in the other work because this code is supposed to work with particles of a radius up to 250nm (see Ref. [11] Section 9.1.2 and Ref. [12] Section 2.1.4.a). The calculations regarding Mie scattering are more complex concerning the ones of Gans scattering and so require more time and CPU, but allow to work without NPs size limitation, and the calculations are computed only once, lightening the computational burden in the long term. As compared to Machine Learning [16], this Monte Carlo approach allows to extract a size distribution and also results in being faster than a classical gradient descent calculation both in terms of time and computational burden. Using gradient descent, every parameter update will contain a sum over all the radii and all the wavelengths will be iterated *m* times as follows:

$$\theta_{j}:=\theta_{j}-\alpha \frac{\partial }{\partial \theta_{j}}ERROR=\theta_{j}-2\alpha \sum_{\lambda_{i}=\lambda_{min}}^{\lambda_{max}}\left(\sigma_{tot}\left(\lambda_{i}\right)-\sigma_{data}\left(\lambda_{i}\right)\right)\frac{\partial }{\partial \theta_{j}}\sigma_{tot}\left(\lambda_{i}\right)=$$

(6) $$=\theta_{j}-2\alpha \sum_{\lambda_{i}=\lambda_{min}}^{\lambda_{max}}\sum_{r_{k}=r_{0}}^{r_{max}}\left(f\left(r_{k},\theta \right)\sigma_{database}\left(\lambda_{i},r_{k}\right)-\sigma_{data}\left(\lambda_{i}\right)\right)\sigma_{database}\left(\lambda_{i},r_{k}\right)\frac{\partial }{\partial \theta_{j}}f\left(r_{k},\theta \right)$$

where α is the learning rate (using the Machine Learning formalism) and this will require at least number_dataset_elements×number_of_wavelengths×m×j operations. The specific operation is described by Equation (6) that contains the derivative of the distribution. This can be evaluated numerically in every cycle or pre-computed, increasing the computational burden. The computational burden of the Monte Carlo fitting depends instead only on some simple mathematical operation (exponentials in distribution evaluation in Equation (2) or (3) and squaring in Equation (4)) and two summations (over the radii in Equation (1) and over the wavelength in Equation (4)) iterated *m* times. The parameter update of Equation (5) recalls only a random number generation, so the computational burden depends linearly on the radius range chosen in the dataset, on the wavelength range of the experimental data, and on the chosen number of iterations. Both methods were tested on the same machine (Intel Core i7 11th Gen, 16 GB RAM, data file 351 elements, radius dataset 500 elements): the Monte Carlo method with 900 iterations was executed in only 45 seconds, while the gradient descent required at least 40 seconds each iteration.

## 3. Results

The experimental extinction spectra of the copper solutions (dotted lines in Figure 5) present the typical copper plasmonic peak at ∼600 nm [4,11] and a strong absorbance in the UV region that goes to zero in the IR. The spectra of copper NPs produced in methanol and ethanol present also a hint of a large shoulder between 600 and 800 nm and a plateau between 400 and 500 nm, suggesting the presence of agglomerates or particles in the order of 100 nm radius. Bimodal distribution is reported to be intrinsic on the PLAL technique [24] and also a previous study on copper NPs [20] confirms this trend, so with these samples, the bimodal distribution was used. Also, the mono distribution fitting was tested, leading, while the mono distribution fitting lead no appreciable results. Copper NPs produced in acetone were fitted with a mono distribution.

**Figure 4:**
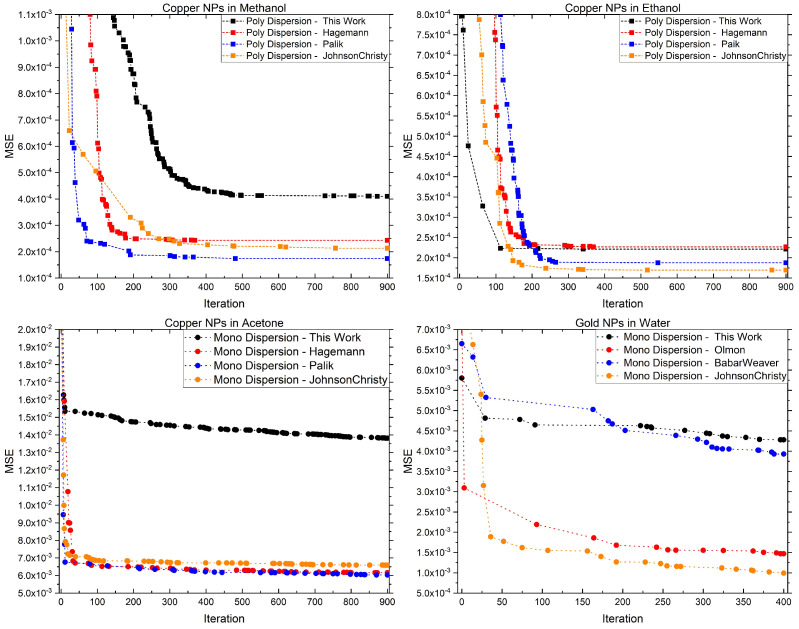
Computed MSE through the various Monte Carlo iteration for copper NPs solution (this work) and gold NPs solution (adapted from Ref. [13]). The points indicate the iteration in which there was a parameter update. The label in the legend indicates both if it refers to a monodispersion or a polydispersion and the used refractive index: “This Work”—Appendix A; “Hagemann”—Refs. [22,26], “Palik”—Ref. [23]; “JohnosnChristy”—Refs. [22,27]; “Olmon”—Refs. [22,28]; “BabarWeaver”—Refs. [22,29]. The fitting routine is iterated 400 times on gold NPs and 900 times on copper NPs. All the fitting results are presented in Appendix D.

The Monte Carlo algorithm was iterated for 400 or 900 cycles. Figure 4 shows how the error rapidly decreases in the first ∼200 steps and then remains stable for each colloidal solution, except for some fine adjustments, proving that the algorithm is fast and converges and justifying the choice to tighten the range at each iteration.

The wavelength range in which the fit is performed and the initial parameter choice are crucial. Fitting tests were conducted down to 200 nm in wavelength, resulting in values $\mu_{1}$ around ∼0.1 nm. This value has no physical meaning because it corresponds to the copper atomic radius [30] and may come from some instrument artifacts in the UV region. For this reason, the fitting range was restricted to 250–1000 nm.

Each graph of Figure 4 represents the MSE behavior of four refractive indexes through the algorithm iterations. The fitting of copper NPs produced in methanol and acetone using the copper refractive index evaluated in this work apparently are the worst, even if the obtained refractive index is very close to the literature ones. These discrepancies in terms of final MSE are very low (0.002 for methanol and 0.01 for acetone) and come from the intrinsic differences of the refractive indexes available in the literature.

**Figure 5:**
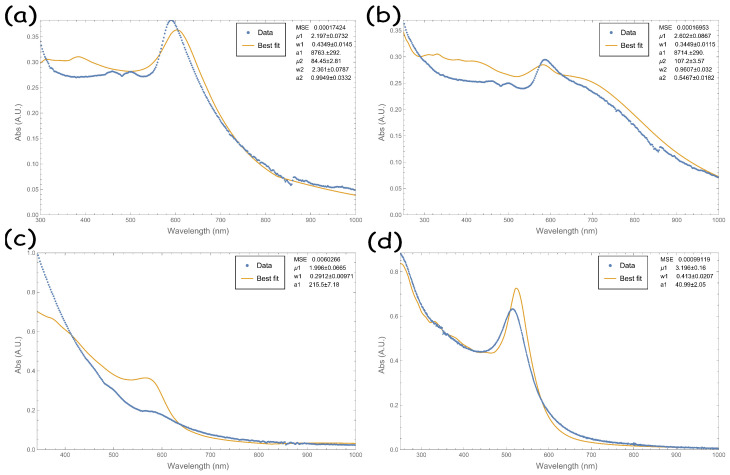
Best-fitting extinction of copper NPs produced in (**a**) methanol, (**b**) ethanol, (**c**) acetone, and (**d**) gold NPs produced in water. In each graph, the blue dots represent the experimental cross section, and the yellow line represents the best fit. In (**a**–**c**), the experimental data are obtained using the methodology described in Section 2.1. In (**d**), the data are adapted from Ref. [13]. The inset reports the fitting MSE and distribution parameters.

Figure 5 and the tables in Appendix D report the obtained best-fitting parameters. The fit curves strongly adapt to the data in the Vis-IR region (λ > 600 nm), while the fit is not satisfying in the Vis-UV region. Despite these discrepancies, the algorithm applied on copper NPs produced in methanol converges on $\mu_{1}∼2.5\;\mathrm{nm},w_{1}∼0.4,\mu_{2}∼85\;\mathrm{nm},w_{2}∼5\;\mathrm{nm}$ with all the used refractive indexes. Copper NPs produced in ethanol results in $\mu_{1}∼2\;\mathrm{nm},w_{1}∼0.3,\mu_{2}∼108\;\mathrm{nm},w_{2}∼2\;\mathrm{nm}$, and copper NPs produced in acetone results in $\mu_{1}∼2.1\;\mathrm{nm},w_{1}∼0.2$.

In Refs. [20,31], the same NPs are produced with slightly the same process parameters and analyzed with TEM, resulting in $\mu_{1}=2.1\;\mathrm{nm},w_{1}=0.62$ for Cu NPs in methanol, $\mu_{1}=3.3\;\mathrm{nm},w_{1}=0.52$ for Cu NPs in ethanol and $\mu_{1}=2.6\;\mathrm{nm},w_{1}=0.14$ for Cu NPs in acetone. For NPs dispersion produced with PLAL, the mean radius value is strongly dependent on the laser fluence, and some casual fluctuations may occur; in fact, the algorithm finds very similar values. The distribution width ($w_{1}$) instead maintains the same trend $w_{Methanol}>w_{Ethanol}>w_{Acetone}$ in both the fitted and measured distributions. For the polydispersed fitted distributions, the ratio $a_{1}/a_{2}$ order of magnitude is $10^{3}$ (methanol) and $10^{5}$ (ethanol), indicating that the number of small particles is greater than that of the bigger ones. The latter majorly contributes to the total cross section, especially in the IR region.

Lastly, the algorithm is tested on the spectra of gold NPs produced in water with PLAL adapted from Ref. [13], obtaining μ=3.4nm,w=0.2 compared to μ=3.5nm,w=0.05 obtained in their work using the same gold refractive index. Using some other works in the literature and measured refractive indexes, the algorithm converges to the values of μ=3nm,w∼0.3.

As the values obtained are comparable, the competitiveness of the developed algorithm, for copper NPs or other metals, is validated. However, the percentage error associated with the parameters (less than 5% for more than 400 iterations) is an underestimation. The main source of error is not the precision of the algorithm but the refractive index itself. The final values of size and distribution spread (μ and *w* in Tables of Appendix D). can be comparable to each other independent of the used refractive index if an error of 10% is considered. This value has no catastrophic consequence for the possible applications (for example, an uncertainty of 0.2nm for the value of 2nm of the distribution peak) and proves the algorithm’s robustness against the biggest source of error.

## 4. Conclusions

**Figure A1:**
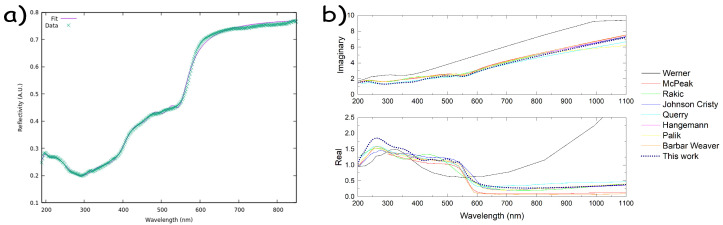
(**a**) Copper reflectivity measured and fitted. (**b**) Copper refractive index from the literature with the addition of the one in this work.

The need to create a versatile instrument that can be easily adapted to every metallic material, changing a few operational parameters, is not expected to produce a perfect fit for several reasons: (i) The starting point is the particle refractive index, but for each material, many different refractive indexes are available in the literature in different wavelength ranges and resolutions as seen in the case of copper in Figure A1 (Appendix A). (ii) A potential layer of coating material (adsorbed solvent, surfactants, oxides, …) is not considered. (iii) Mie scattering theory was used in this work. This theory is related only to spherical particles, while Gans theory provides information on spheroidal particles only in the quasi-static limit. NPs can assume complicated shapes, depending on the production technique, but still, the sphere remains the best approximation. An ideal scenario is the one with spherical particles of the same size, but the reality usually is far away, and the algorithm applied on the spectrum of elongated structures with a high aspect ratio or sharp edges can produce results with no physical significance. (iv) The dielectric correction due to the small size of nanoparticles (Appendix C, Equations (A9) and A10)) is not unique: many are proposed, and the most generic one for a sphere [12] is used. Also, parameters such as the plasma frequency $\omega_{p}$, the electron Fermi velocity $v_{F}$, and the electron mean free path $l_{\infty }$ are not univocal in the literature for the studied material [12,32,33].

The algorithm was tested on metallic NPs but can ideally work also with semiconductor or insulating material. In this case, the correction described in Appendix C involving Equations (A9) and (A10) can be neglected.

Nonetheless, a small variation of these parameters or refractive index produces a small variation of the simulated cross sections, but the general trend remains unchanged. So, even if the fitted distribution apparently differs from the experimental data in some wavelength ranges, the obtained distribution parameters are reliable. This makes the proposed approach useful for extracting information from a simple and quick optical measurement.

## Figures and Tables

**Figure A2:**
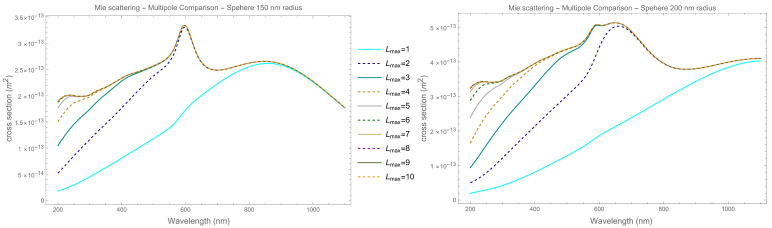
Computed extinction cross section for particle of 150 nm and 200 nm radius truncated at different multipole order ($L_{max}$).

## Data Availability

All the codes, the experimental data, the dataset computed, and the refractive index files mentioned in this work are available on GoogleDrive at the following link: https://drive.google.com/drive/folders/1oIPeAoi8S0_q63F3alHogbICgwl--KxU?usp=sharing accessed on 27 October 2023.

## Abbreviations

    The following abbreviations are used in this manuscript:

NPs	Nanoparticles
PLAL	Pulsed Laser Ablation in Liquid
MSE	Mean Squared Error
SEM	Scanning Electron Microscopy
TEM	Transmission Electron Microscopy

## Appendix A. Copper Refractive Index

The copper refractive index used for this work was obtained by fitting the reflectance spectrum of copper, using as a sample the same target used for PLAL polished with isopropyl alcohol and rinsed with $N_{2}$.

Fitting was performed using Gnuplot 5 software [34] in the wavelength range 190–850 nm, using a Drude dielectric function:

(A1) $$\epsilon \left(\omega \right)=\epsilon_{0}+\frac{\omega_{p}^{2}}{-\omega^{2}-i\Gamma \omega }+\sum_{j}\frac{\omega_{p_{j}}^{2}}{\omega_{0_{j}}^{2}-\omega^{2}-i\Gamma_{j}\omega }$$

The reflectivity then was obtained as follows:

(A2) $$\lambda \left[nm\right]=\frac{1239[\mathrm{eV}/\mathrm{nm}]}{\omega [\mathrm{eV}]}\;\;n\left(\lambda \right)=\sqrt{\epsilon (\lambda )}\;\;R={\left|\frac{n(\lambda )-1}{n(\lambda )+1}\right|}^{2}$$

The free electron oscillator (the one outside the sum in Equation (A1)) was forced with $\omega_{p}=8.76\;\mathrm{eV}$ and $\Gamma =95.5\cdot 10^{-3}\;\mathrm{eV}$ [32,35]. Then $\epsilon_{0}$ and the parameters of seven additional oscillators were fitted. The resulting refractive index is comparable to the one reported in literature [22] as seen in Figure A1. The same procedure was repeated to obtain the gold refractive index, using the free electron oscillator forced with $\omega_{p}=8.89\;\mathrm{eV}$ and $\Gamma =70.88\cdot 10^{-3}\;\mathrm{eV}$ [32,35] and four additional oscillators.

## Appendix B. Solvent Refractive Indexes

Usually, organic solvents have a constant refractive index in the Vis-IR range that strongly increases in the UV range. Formulas for the used solvent refractive index are taken from the online refractive index database [22]. In particular, the methanol refractive index [36]:

(A3) $$n=1.3195+3.05364419\cdot 10^{-3}\lambda^{-2}-3.41636393011\cdot 10^{-5}\lambda^{-4}+2.62128\cdot 10^{-6}\lambda^{-6}$$

Ethanol refractive index [36]:

(A4) $$n=1.34959+4.0147128\cdot 10^{-3}\lambda^{-2}-5.9411155\cdot 10^{-5}\lambda^{-4}+3.04975\cdot 10^{-6}\lambda^{-6}$$

Acetone refractive index [37]:

(A5) $$n=1.34979+0.00306\lambda^{-2}+0.00006\lambda^{-4}$$

Water refractive index is taken from a list of points [22,38].

## Appendix C. Mie Scattering cross Section Simulation

The file Dataset_creation.nb is structured as follows.

First, Riccati–Bessel functions are defined as:

(A6) $$\psi_{L}\left(x\right)=x\sqrt{\frac{\pi }{2x}}BesselJ\left(L+\frac{1}{2},x\right)$$

(A7) $$\eta_{L}\left(x\right)=x\sqrt{\frac{\pi }{2x}}\left(BesselJ\left(L+\frac{1}{2},x\right)+iBesselY\left(L+\frac{1}{2},x\right)\right)$$

where BesselJ and BesselY are the Bessel function [39]. Then, two functions are defined:

(A8) $$k\left(\lambda \right)=\frac{2\pi }{\lambda }\;\omega \left(\lambda \right)=k\left(\lambda \right)c$$

where *c* is the speed of light. The following part is repeated in a cycle for every radius in a range (from 0.5 nm to 200 nm, 0.5 nm step is used, and from 0.1 nm to 30 nm, 0.1 nm step is used). At the cycle beginning, the correction to the dielectric function is applied [12]:

$$\epsilon \left(\lambda \right)=n{\left(\lambda \right)}^{2}$$

(A9) $$\epsilon :=\epsilon +\omega_{p}^{2}\left(\frac{1}{\omega^{2}+\Gamma_{B}^{2}}-\frac{1}{\omega^{2}+\Gamma {\left(R\right)}^{2}}\right)+i\frac{\omega_{p}^{2}}{\omega }\left(\frac{1}{\omega^{2}+\Gamma {\left(R\right)}^{2}}-\frac{1}{\omega^{2}+\Gamma_{B}^{2}}\right)$$

$$n\left(\lambda \right):=\sqrt{\epsilon (\lambda )}$$

where $\omega_{p}$ is the plasma frequency (Cu:8.76, Au:8.89[eV] [32,35]), and the dumping frequency $\Gamma_{B}=v_{f}/l_{\infty }$ is defined as the ratio between the electron Fermi velocity $v_{f}$ (Cu:$1.57\cdot 10^{6}$, Au:$1.40\cdot 10^{6}$[m/s] [33,40]) and the electron mean free path $l_{\infty }$ (Cu:42, Au:42 $\left[10^{-9}m\right]$ [12]). The new dielectric function is size dependent with Γ(R):

(A10) $$\Gamma \left(R\right)=\Gamma_{B}+A\frac{v_{f}}{R}$$

where *R* is the particle radius and A=3/4 [12]. Then, the cross section is evaluated in a cycle for each wavelength within a range (the used one is from 250 nm to 1000 nm, 1 nm step). The ratio between the refractive index of the nanoparticles n(λ) and the medium nm(λ) is computed at a given wavelength, and so the adimensional quantity *X*:

(A11) $$m=\frac{n(\lambda )}{nm(\lambda )}\;\;\;X=k\left(\lambda \right)R$$

where *R* is the nanoparticle radius (in nanometers). Then, two functions are computed:

(A12) $$a_{L}=\frac{m\psi_{L}\left(mX\right)\psi_{L}^{\prime }\left(X\right)-\psi_{L}^{\prime }\left(mX\right)\psi_{L}\left(X\right)}{m\psi_{L}\left(mX\right)\eta_{L}^{\prime }\left(X\right)-\psi_{L}^{\prime }\left(mX\right)\eta_{L}\left(X\right)}$$

(A13) $$b_{L}=\frac{\psi_{L}\left(mX\right)\psi_{L}^{\prime }\left(X\right)-m\psi_{L}^{\prime }\left(mX\right)\psi_{L}\left(X\right)}{\psi_{L}\left(mX\right)\eta_{L}^{\prime }\left(X\right)-m\psi_{L}^{\prime }\left(mX\right)\eta_{L}\left(X\right)}$$

And finally, the cross sections are computed:

- The scattering cross section: $\sigma_{sca}=\frac{2\pi }{k{\left(\lambda \right)}^{2}}\sum_{L=1}^{L_{max}}\left(\right|a_{L}{|}^{2}+|b_{L}{|}^{2})$.
- The extinction cross section: $\sigma_{ext}=\frac{2\pi }{k{\left(\lambda \right)}^{2}}\sum_{L=1}^{L_{max}}\left(2L+1\right)Re\left(a_{L}+b_{L}\right)$.
- The absorption cross section: $\sigma_{abs}=\sigma_{ext}-\sigma_{sca}$.

The cross sections are defined by a sum over multipole *L* up to $L_{max}$. The cross section calculated on small particles (radius < 50 nm) converges with just $L_{max}=3$, while on bigger particles (radius up to 250 nm), at least $L_{max}=10$ is necessary to converge, especially in the UV as reported in Figure A2. In this work, $L_{max}=\left(2\left(Integer\_part\left[R/50\right]+1\right)+1\right)$ is used, so the multipole order starts from $L_{max}=3$ for smaller particles and increases by 2 every 50 nm radius. This approach is more precise but requires a lot of computational time, especially with bigger particles. At the end of the cycle, the dataset file is exported.

## Appendix D. Results of Fitting with Different Refractive Indexes

**Table A1:** Best-fitting parameters and MSE copper NPs produced in methanol, dataset computed with various refractive indexes and various radius ranges.

Particle Refractive Index	Dataset Range [nm] (Start:Step:Stop)	Parameter Error	MSE	a1	μ1 [nm]	w1	a2	μ2 [nm]	w2 [nm]
This work	0.5:0.5:250	3.3%	0.0004	$3.6\cdot 10^{3}$	3.9	0.289	0.90	84	4.5
[22,26]	0.5:0.5:250	3.3%	0.0002	$4.3\cdot 10^{3}$	2.90	0.37	1.06	83	8.9
[23]	0.5:0.5:250	3.3%	0.0002	$8.8\cdot 10^{3}$	2.20	0.43	0.99	88	2.36
[22,27]	0.5:0.5:250	3.3%	0.0002	$7.2\cdot 10^{3}$	2.48	0.39	1.05	83	5.6
This work	0.1:0.1:30	5%	0.01	$1.25\cdot 10^{4}$	0.50	0.0.25			
[22,26]	0.1:0.1:30	5%	0.01	$1.12\cdot 10^{3}$	1.13	0.150			
TEM distribution from Ref. [20]					2.1	0.62			

**Table A2:** Best-fitting parameters and MSE copper NPs produced in ethanol, dataset computed with various refractive indexes and various radius ranges.

Particle Refractive Index	Dataset Range [nm] (Start:Step:Stop)	Parameter Error	MSE	a1	μ1 [nm]	w1	a2	μ2 [nm]	w2 [nm]
This work	0.5:0.5:250	3.3%	0.0002	$5.9\cdot 10^{4}$	1.37	0.37	0.53	108	4.2
[22,26]	0.5:0.5:250	3.3%	0.0002	$4.2\cdot 10^{4}$	1.63	0.31	0.53	108	2.43
[23]	0.5:0.5:250	3.3%	0.0002	$8.8\cdot 10^{3}$	2.20	0.43	0.99	88	2.36
[22,27]	0.5:0.5:250	3.3%	0.0002	$8.7\cdot 10^{3}$	2.60	0.087	0.55	107	0.96
This work	0.1:0.1:30	5%	0.01	310	1.50	0.22			
[22,26]	0.1:0.1:30	5%	0.01	$1.19\cdot 10^{3}$	0.99	0.20			
TEM distribution from Ref. [20]					3.3	0.52			

**Table A3:** Best-fitting parameters and MSE copper NPs produced in acetone, dataset computed with various refractive indexes and various radius ranges.

Particle Refractive Index	Dataset Range [nm] (Start:Step:Stop)	Parameter Error	MSE	a1	μ1 [nm]	w1
This work	0.1:0.1:30	3.3%	0.01	128	2.32	0.225
[22,26]	0.1:0.1:30	3.3%	0.006	172	2.15	0.288
[23]	0.1:0.1:30	3.3%	0.006	216	2.00	0.291
[22,27]	0.1:0.1:30	3.3%	0.007	294	1.96	0.227
TEM distribution from Ref. [31]					2.6	0.14

**Table A4:** Best-fitting parameters and MSE gold NPs produced in Water (data adapted from Ref. [13]), dataset computed with various refractive indexes and various radius ranges.

Particle Refractive Index	Dataset Range [nm] (Start:Step:Stop)	Parameter Error	MSE	a1	μ1 [nm]	w1
This work	0.1:0.1:30	5%	0.004	90	3.0	0.20
[22,28]	0.1:0.1:30	5%	0.001	72	3.4	0.196
[22,29]	0.1:0.1:30	5%	0.004	119	2.8	0.32
[22,27]	0.1:0.1:30	5%	0.001	41	3.2	0.41
Fitted with code adapted from Ref. [13]					3.5	0.05
